# The role of gut microbiota in liver regeneration

**DOI:** 10.3389/fimmu.2022.1003376

**Published:** 2022-10-27

**Authors:** Zhe Xu, Nan Jiang, Yuanyuan Xiao, Kefei Yuan, Zhen Wang

**Affiliations:** ^1^ Department of Liver Surgery, State Key Laboratory of Biotherapy and Cancer Center, West China Hospital, Sichuan University and Collaborative Innovation Center of Biotherapy, Chengdu, China; ^2^ Laboratory of Liver Surgery, State Key Laboratory of Biotherapy and Cancer Center, West China Hospital, Sichuan University and Collaborative Innovation Center of Biotherapy, Chengdu, China; ^3^ Department of Obstetrics and Gynecology, West China Second Hospital of Sichuan University, Chengdu, China; ^4^ Key Laboratory of Birth Defects and Related Diseases of Women and Children, Sichuan University, Ministry of Education, Chengdu, China

**Keywords:** liver regeneration, gut microbiota, microbial metabolic activity, regulate gut microbiota, inflammatory cytokines

## Abstract

The liver has unique regeneration potential, which ensures the continuous dependence of the human body on hepatic functions. As the composition and function of gut microbiota has been gradually elucidated, the vital role of gut microbiota in liver regeneration through gut-liver axis has recently been accepted. In the process of liver regeneration, gut microbiota composition is changed. Moreover, gut microbiota can contribute to the regulation of the liver immune microenvironment, thereby modulating the release of inflammatory factors including IL-6, TNF-α, HGF, IFN-γ and TGF-β, which involve in different phases of liver regeneration. And previous research have demonstrated that through enterohepatic circulation, bile acids (BAs), lipopolysaccharide, short-chain fatty acids and other metabolites of gut microbiota associate with liver and may promote liver regeneration through various pathways. In this perspective, by summarizing gut microbiota-derived signaling pathways that promote liver regeneration, we unveil the role of gut microbiota in liver regeneration and provide feasible strategies to promote liver regeneration by altering gut microbiota composition.

## Introduction

### Liver regeneration

Liver is the largest substantial organ in the human body, it composed of parenchymal cells (hepatocytes) and non-parenchymal cells (endothelial cells, Kupffer cells, lymphocytes, and stellate cells). Different from other tissues and organs in our body, normal liver has powerful regenerative potential to maintain an appropriate size relative to the rest of the body. Hepatocytes spend most time in the G0 phase of the cell cycle and thus rarely divide normally (1). However, after injury or excision, the proliferative potential of hepatocytes is activated. Macroscopically, it is expressed as the remaining liver undergoes a rapid series of compensatory hyperplasia to regain its original volume and structure and meet the metabolic needs of the organism (1, 2), which is called liver regeneration. It is worth noting that the regeneration is not referred to excised parts regeneration, but the remaining liver expands massively to compensate for lost tissue (3).

The classic model of liver regeneration, the two-thirds partial hepatectomy (PH) rat model first proposed by Higgins et al. in 1931 (4), has been studied for decades. And the detailed mechanisms are being studied (5, 6). Decades of studies revealed that liver regeneration is a complex network activated by multiple pathways. To summarize, liver regeneration can be divided into three phases: initiation, proliferation and termination. Pro-inflammatory cytokines tumor necrosis factor α (TNF-α) and Interleukin 6 (IL-6) mediate the priming phase (2, 7, 8).

**Figure 1:**
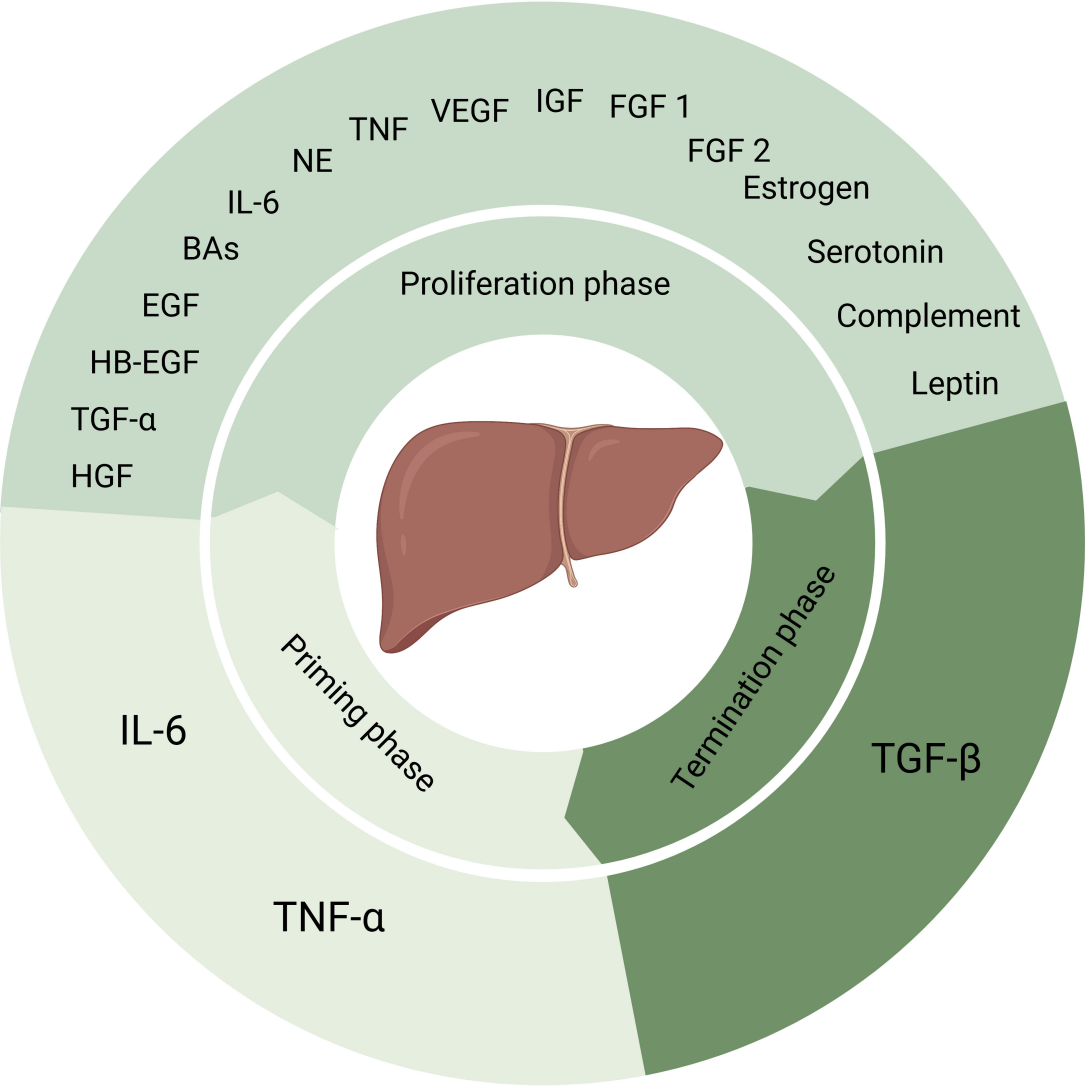
Factors that play a role in process of liver regeneration. IL-6 and TNF-α induce the priming phase. Complete mitogens including HGF, EGF, HB-EGF and TGF-α, together with auxiliary mitogens BAs, norepinephrine, TNF, IL-6, vascular endothelial growth factor, insulin-like growth factor system, estrogen, serotonin, leptin, complement, FGF1 and FGF2 involve in the proliferation phase. And the termination phase is induced by TGF-β. NE, norepinephrine, VEGF, vascular endothelial growth factor, IGF, insulin-like growth factor.

Kupffer cells are main source of TNF-α and IL-6. And the release of TNF-α and IL-6 through the NF-κB signaling pathway is triggered either by gut microbial lipopolysaccharide (LPS)/Toll-like receptor 4 (TLR4) signaling or by C3a and C5a (7, 9). The second phase is the proliferation phase converting cells from G1 phase to mitosis mainly mediated by complete mitogens HGF, epidermal growth factor (EGF), heparin-binding EGF-like growth factor (HB-EGF), and transforming growth factor-α (TGF-α) (2, 7, 8, 10). And auxiliary mitogens bile acids (BAs), norepinephrine, TNF, IL-6, vascular endothelial growth factor (VEGF), insulin-like growth factor system, estrogen, serotonin, leptin, complement, fibroblast growth factor 1 (FGF1) and FGF2 promote mitosis (2, 7, 8). The absence of these auxiliary mitogens may delay but not eliminate liver regeneration. When the normal liver mass/body mass ratio of 2.5% has been restored, the termination phase would be started. Past research suggested that TGF-β plays a major role in this phase (2, 7, 8, 10). Yet, more evidence needs to be added in this phase (**Figure 1**).In clinical practice, it is common for patients to receive PH due to liver trauma, liver malignancy, liver hydatid disease, cirrhosis and many other liver diseases. And in patients who have received PH, remnant liver regeneration is slow and liver failure is common due to individual differences. Although liver transplantation is an effective treatment approach, the shortage of donors severely limits its application. Therefore, in order to improve the outcome of patients after liver surgery, it is necessary and urgent to study the molecular regulatory mechanism of liver regeneration, discovering potential regulatory target molecules, and exploring new therapeutic strategies to improve the regeneration ability of remnant liver after hepatectomy to restore its function quickly.

With the development of metabolomics, emerging studies have used the integrating metabolomics to characterize the metabolic rewiring of hepatocytes in proliferation and liver regeneration. It has been reported that proliferating liver tissue had distinct lipid profiles to their corresponding control group for all regeneration models. The level of monounsaturated fatty acid (MUFA)–containing phosphatidylcholine (PC), phosphatidylethanolamine (PE), short chain triacylglycerides (TAGs), and free cholesterol have increased, with a decrease in polyunsaturated fatty acid (PUFA)–containing PCs and sphingomyelin (SM) (11). Also, the mitochondrial oxidation has unique characteristics during liver regeneration, mainly concerning the level of NADH changed (12). With the knowledge of metabolomics profiles of liver regeneration increasing, we can use clinical metabolomics combined with machine learning algorithms to predict liver regeneration (13).

Besides, the gut microbial metabolites have also been proved to be the important molecules in the liver regeneration. BAs involve in the proliferation phase of hepatocytes and can be reabsorbed through enterohepatic circulation from gut. But the primary BAs overload in liver in turn inhibits liver regeneration. And the short chain fatty acid (SCFA), which is the product of metabolism in gut, is beneficial to liver metabolism homeostasis, improving liver generation. Besides these two major metabolites, hydrogen, indoles and its derivatives can also improve liver regeneration by enhancing gut epithelial barrier.

Finding the clinical intervention methods of multi liver diseases aiming to promote liver regeneration related metabolism by modulating gut microbiota is a promising future research area. In addition, inhibited liver regeneration has been observed in the process of many liver diseases (14). It is not conducive to the repair of the patient’s liver. Therefore, finding out related promoting factors of liver regeneration has a great significance in clinical treatment of liver diseases.

### Gut microbiota

Gut microbiota is a unique array of bacteria and other microorganisms located in the human gastrointestinal tract, which is the largest symbiotic ecosystem with the host (15). The total estimated number of gut microbiotas is somewhere between 10^13^ and 10^14^ (16). Human gut bacteria are mainly from *Firmicutes* (60 to 80%), *Bacteroidetes* (20 to 40%), *Proteobacteria*, *Actinobacteria*, *Verrucomicrobia*, *Fusobacteria*, and *Cyanobacteria* (17). The related proportion of these bacterial phyla has been proved to affect multiple dimensions of human health, including liver regeneration (18, 19). In view of its huge impact on health, the idea of considering gut microbiota as a “virtual metabolic organ” or”previously forgotten organ” has been proposed in recent years.

Gut microbiota affects liver regeneration after liver injury through the gut-liver axis. The gut-liver axis is the bridge that links the human intestine to liver, and it is a consequence of a close anatomical and functional, bidirectional interaction of the intestine and liver (20). On the one hand, the liver releases BAs and many bioactive mediators into the biliary tract and the systemic circulation (21). And then these bioactive mediators and BAs arrive in intestine and perform functions. On the other hand, metabolites produced by host or gut microbiota and exogenous substrates in the intestine translocate to the liver through portal vein and influence liver functions. In gut-liver axis, the intestinal barrier controls the transport from the gut to the liver, it includes gut mucosal epithelial barrier and epithelial physical barrier. The gut mucosal epithelial barrier is the largest mucosal surface in human body, covering approximately 400 m^2^ of surface area with a single layer of intestinal epithelial cells (22). This barrier limits the translocation of microbiota and some metabolites from the intestine, while allowing active transport of nutrients through tight junction. Therefore, appropriate permeability of this barrier is part of the gut-liver axis. In many pathological processes, metabolites and bacterial translocation is increased with the increased permeability of gut mucosal epithelial barrier (23), and this leads to many liver injury and inhibits liver regeneration. Epithelial physical barrier function is mediated by a series of intercellular junctions including apical tight junction, subjacent adherents junction and desmosomes (24). Other factors, such as mucins, antibacterial peptides, immunoglobulins, intraepithelial lymphocytes, also contribute to enhance the barrier function (21, 25). Intestinal inflammation, often accompanied by dysregulation of the gut microbiota, can increase the permeability of gut mucosal epithelial barrier and has been observed in many liver diseases (24, 26). Gut microbiota and ite metabolites affect the metabolism and secretion of cytokines of cells in gut and liver. Thus, they manage to promote liver regeneration (22, 26).

Metabolites and the immune regulation are two main factors affecting liver regeneration through gut microbiota. The change of the proportion of gut microbiota can effectively regulate the above two factors and thus affect liver regeneration. Multiple methods, such as probiotics, prebiotics and antibiotics can be used to adjust the proportion of gut microbiota and thus, promote liver regeneration.

## Gut microbiota composition changes during liver regeneration

Studies investigated changes in gut microbiota composition during liver regeneration after PH in mice and rats respectively.

In the study of mice, liver cell proliferation started 1 day after PH, peaked on day 2, and ceased on day 9 (19). The most two abundant phyla, *Bacteroidetes* and *Firmicutes*, showed a completely different trend in the number of the changes. *Bacteroidetes* abundance steadily increased while *Firmicutes* reciprocally decreased during liver regeneration. At family level, increased S24-7 and *Rikenellaceae* caused *Bacteroidetes* expansion while decreased *Clostridiales*, *Lachnospiraceae*, and *Ruminococcaceae* caused *Firmicutes* contraction. Above changes in the composition of the gut microbiota were observed consistently over 9 days after PH, which includes the priming phase, proliferative phase, and termination phase of liver regeneration (19).

In the study of rats, liver cell proliferation started 30 h after PH, peaked at 48 h, and was terminated by 168 h (27). The abundance of *Bacteroidetes* decreased at 12 h after PH, but steadily increased to normal level at 48h. Then the abundance of *Bacteroidetes* decreased on day 3 and maintained a low level until the end of observation. However, the abundance of *Firmicutes* was inversely changed. At family level, *Lachnospiraceae* and *Ruminococcaceae*, which are the most abundant taxa within the Firmicutes phylum, increased during 12~24 hours and 3~14 days after PH, but decreased during 30~48 hours. In addition, the abundance of *Proteobacteria* increased remarkably during the first 48h after PH (27).

Besides, in a study that retinoic acid accelerated liver regeneration in mice, the authors report that there is a reduced ratio of *Firmicutes* to *Bacteroidetes* 1 day after PH in retinoic acid-treated mice (28). At family level, the abundance of *Ruminococcaceae* decreased from day zero to day 1 after PH and increased from day 1 to day 2. And the abundance of *Lachnospiraceae* increased during the first half day and from day 1.5 to day 2 after PH and decreased from day 0.5 to day 1.5. In addition, the abundance of *Bifidobacterium* was also dramatically higher in retinoic acid-primed mice on day zero and day 1 after PH (28) (**Table 1**).

**Table 1:** The variation trend of different type of gut microbiota after PH.

Type	Variation trend after PH	Subtype	Variation trend after PH	References
Bacteroidetes	↑	S24-7	↑	(24)
		Rikenellaceae	↑	
		/	/	(26)
	↓	S24-7	↓	(25)
		Bacteroidia	↑	(25)
		Prevotellaceae	↓ after PH till day2,↑from day2 to day14	
Firmicutes	↑	Ruminococcaceae	↓ after PH till day2,↑from day2 to day14	(25)
		Lachnospiraceae	↓ after PH till day2,↑from day2 to day14	(25)
	↓	Ruminococcaceae	↓	(24, 25)
			↓from day zero to day1,↑from day 1 to day 2.	(26)
		Lachnospiraceae	↓	(24)
			↑from day0 to day0.5 and from day 1.5 to day 2,↓from day 0.5 to day 1.5	(26)
		Clostridiales	↓	(24, 25)
		/	/	(26)
Proteobacteria	↑	/	/	(25)
Actinobacteria	↑	Bifidobacterium	↑	(26)

↑ refers to upward trend; ↓ refers to downward trend.

The results of these studies are not exactly the same, which may be caused by different research objects and experimental designs. Although more studies are needed to investigate changes in gut microbiota composition during liver regeneration, we can predict that the changes in gut microbiota composition may be the result of adaptation after PH and the composition changes may promote liver regeneration by altering gut mucosal epithelial barrier permeability, influencing metabolite release and many other pathways.

## Gut microbiota affects liver regeneration by modulating crucial cytokines in regeneration

### IL-6

While IL-6 is associated with many liver pathologies and cancers, IL-6 also plays an important role in liver regeneration. And IL-6 level has also been used as a secondary endpoint in many studies concerning liver regeneration. The priming phase of the liver regeneration is mediated by TNF-α and IL-6. IL-6 mediates around 40% of liver regeneration related gene expression (29). IL-6 promotes liver regeneration through inducing hepatocyte proliferation. IL-6 binds to an 80 kDa IL-6 receptor on its target cells and then the complex of IL-6 and IL-6 receptor associates with a second receptor protein, glycoprotein(gp) 130 (30). The cytoplasmic portion of gp130 dimer associates with JAKs and active the JAK/STAT pathway (31, 32). Later, STAT3 dimer translocates into the nucleus and activates regeneration and mitosis related early genes, promoting transition of the quiescent hepatocytes from G0 phase into G1 and S phases (33). Besides, IL-6 can induced tyrosine phosphorylation of gp130, tyrosine phosphorylated gp130 can mediate the activation of the Ras-Raf-MAPK signaling pathway together with JAK1 (32). IL-6 or IL-6 receptor alone cannot associate with gp130. While gp130 is expressed on all cells, the IL-6 receptor is expressed only on some leukocytes and liver cells, including Kupffer cells, hepatocytes, hepatic stellate cells, sinusoidal and endothelial cells (34).

Besides, IL-6 increases anti-apoptotic factor Mcl-1 expression to promote anti-apoptotic effects (35). IL-6 increased the expression of angiotensinogen, which is essential in TNF-α/NF-κB-mediated liver regeneration (36). Also an IL-6 dose-dependent increase in HGF was found in cancer patients (37). And statistically, the number of postoperative complications is negatively correlated with IL-6 after hepatectomy (38). IL-6 stimulation can promote biliary epithelial cell proliferation and activate them to differentiate into hepatic-progenitor cells. The progenitor cells can further differentiate into hepatocytes to innate the liver regeneration (39). These experimental data above pointed to an important role of IL-6 in liver regeneration.

The level of TNF-α and IL-6 have been found up-regulate in the liver vein after hepatectomy (40) and in Associating Liver Partition and Portal Vein Ligation for Staged Hepatectomy(ALPPS) at the mRNA and protein level (41). It is reported that enterogenic LPS can promote the release of IL-6 in Kuppfer cells by acting on TLR4 or through a TNF-α dependent pathway, which involves the involvement of C3 and C5 (42). And the microbiota-depletion mice shows IL-6 considerably reduction in some studies. Moreover, IL-6 knock-out mice show impaired liver regeneration (43). *Odoribacter splanchnicus*, *Bacteroides* sp. *4_1_36*, *Bacteroides* sp. *D20*, and *Bacteroides uniformis* all four strains can stimulate IL-6 producing (44). Some Syk-kinase-coupled C-type lectin receptors (CLRs), such as Dectin-1 (Clec7a), Dectin-2 (Clec4n), and Mincle (Clec4e), preferentially induce myeloid IL-6 by promoting Th17 polizartion and differention. And the mucosa-associated commensals mediated singaling Mincle and Syk pathways in CD11b^+^ dome DCs and lysozyme-expressing DCs (LysoDCs) from Peyer’s patches (PPs) promote the IL-6 secretion in these cells. And the IL-6 production by CD19^-^CD11c^+^MHCII^+^ DCs in PPs are also in Mincle dependent manner, producing activity diminished in Mincle-deficient mice (45).

However, in some related reports of gut microbiota improving liver injury, the function of gut microbiota was described to decrease IL-6. In NAFLD patients receiving multi-probiotic “Symbiter” composed of *Bifidobacterium*, *Lactobacillus*, *Lactococcus*, and *Propionibacterium*, IL-6 together with fatty liver index decreased (46, 47). Similar result was observed in NAFLD patients receiving omega-3 fatty acid (48). Another report showed that receiving probiotics before and after liver transplantation can reduce the levels of IL-6 and other chronic inflammatory mediators, maintain gut microbiota homeostasis, and improve the prognosis of liver transplantation (49).

Although the mechanism of IL-6 affecting liver regeneration has been clearly studied, there are still few studies on the detailed biological process in the regulating effect of gut microbiota on IL-6 and thus promoting liver regeneration, and most reports remain to recognize IL-6 as a secondary endpoint to describe the improvement of gut microbiota on liver disease. More studies are needed to confirm the role of gut microbiota in regulating IL-6 to promote liver regeneration.

### TNF-α

TNF-α promote liver regeneration by mediating the priming phase of the liver regeneration. TNF-α released by Kupffer cells activate TNF receptor 1 on the surface of Kupffer cells in autocrine fashion, up-regulating NF-κB and activating the transcription of IL-6 (50). Besides, TNF-α activate c-Jun N-terminal kinase (JNK) and MAPK-ERK, the crucial regulators of Jun activation and the expression of cyclin D1, a crucial promoter of the hepatocyte cell cycle (50). TNF-α can up-regulate the activity of various homo- and heterodimeric AP-1 transcription factors, and the activation of some proteins involved in growth response requires AP-1 activity (51).

TNF-α is mainly released by Kupffer cells in liver and some can be secreted in intestine CaCO-2 cells were stimulated with non-pathogenic bacteria and enteropathogenic *Escherichia coli*, and level of TNF-α increase. And the release of TNF-α is influenced by gut microbiota. Gut microbiota-derived TLR agonists, such as LPS acts on its receptor TLR4 and TLR9 on Kupffer cells and then recruits and activates MyD88 (myeloid differentiation factor 88), promoting the release of pro-inflammatory factors through NF-κB signaling pathway, including TNF-α (7, 52). Moreover, enterogenic LPS can induce the transformation of C3 and C5 into the bioactive peptides C3a and C5a, both of which act on its receptor on Kupffer cells and induce the release of TNF-α (42). C3-deficient mice and C5-deficient mice showed decreased TNF-α release level and delayed liver regeneration after PH and liver regeneration was inhibited on a higher degree in combined C3/C5-deficient mice (53, 54). Delayed liver regeneration observed in C5-deficient mice was mainly due to a significant delay in liver cell re-entry into the cell cycle (S phase), which is consistent to some extent with TNF-α mediated initiation of liver regeneration (53).

The role of TNF-α is essential in both liver injury and regeneration, but most of the research are focus on the inhibiting effect of gut microbiota towards TNF-α for ameliorating inflammation in pathlogical condition. The role of microbiota in promoting liver regeneration by TNF-α secretion require more extensive research.

### HGF

Hepatic growth factor (HGF) induces hepatocyte DNA synthesis and mitosis in the proliferation phase as complete mitogens.

HGF is secreted mainly by hepatic stellate cells, vascular endothelial cells and Kupffer cells. HGF is a ligand for the tyrosine kinase receptor c-Met (55–57). Activation of HGF/Met axis activate several downstream pathways including ERK1/2, JAK/STAT3, PI3K/AKR/NF-κB, MAPK and Ras/Raf (55, 57, 58). These pathways play a vital role in promoting liver regeneration. HGF receptor was activated in high levels 0.5h after PH, and the level of HGF decreased 0~3h after PH (55). HGF increased to a significantly high-level 3~48h after PH and was inactivated by combination with TGF-β in the termination phase (55, 59). C-Met-deficient mice showed a higher mortality rate after 70% PH (60), suggesting the function of HGF/Met in liver regeneration. *Listeria* monocytogenes, a pathogenic bacterium can be found in various kinds of food, can enhance the effect of HGF through producing bacterial HGF receptor agonist InlB321/15 and thus promote liver regeneration after 70% PH (61). Lactiplantibacillus plantarum AR113 administration showed decline of phosphatidylethanolamine (PE), phosphatidylcholine (PC), phosphatidyl serine (PS), and lysophosphatidyl choline (LysoPC) levels in the serum of the rats and increase HGF secretion in liver after 70% PH (62). Although the correlation of gut microbiota and HGF has been well addressed, the detailed mechanism is need to be further investigated.

### IFN-γ and TGF-β

IFN-γ is produced mainly by activated T cells and natural killer cells. IFN-γ inhibits liver regeneration by inhibiting hepatocyte cycle and inducing hepatocyte apoptosis (63, 64). IFN-γ-induced hepatocyte apoptosis likely involves multiple pathways, including a p53-independent, IRF-1-dependent mechanism, increased production of reactive oxygen species and endoplasmic reticulum stress (63). And IFN-γ inhibits hepatocyte DNA synthesis through inhibiting G1 cell cycle, which requires the involvement of p53 and STAT1 (65). Mice with injection of IFN-γ showed inhibited liver regeneration after partial hepatocyte, but disinhibition of liver regeneration was found in IFN-γ-deficient mice (66). The gut microbiota homeostasis is depend on he IFN-γ-STAT1/STAT3 signaling pathways to improve liver injury and thus promote liver regeneration (67).

Tryptophan-derived metabolites of gut microbiota can activate AhR, and activated AhR increases the release of IFN-γ (68). *F. nucleatum* subsp. *polymorphum* releases outer membrane vesicles which activate TLR4 and NF-κB to stimulate IFN-γ (69). *Christensenellaceae*, *Lactobacillus, B. bifidum, Parabacteroides distasonis* can down-regulate intrahepatic IFN-γ level to promote liver regeneration (25, 70–73). *Bifidobacterium bifidum* was observed to decrease IFN-γ and IL-12 release by NK cell (73). Mice receiving daily oral gavage probiotic compound VSL#3 (*Bifidobacterium longum*, *Bifidobacterium breve*, *Bifidobacterium infantis*, *Lactobacillus casei*, *Lactobacillus plantarum*, *Lactobacillus acidophilus*, *Lactobacillus delbrueckii* subsp, *Bulgaricus*, and *Streptococcus salivarius* subsp) for 7days showed decreased IFN-γ (70). Moreover, mice with oral administration of *Parabacteroides distasonis* antigens also showed decreased IFN-γ compared with control group (71). In both high-salt and high-fat diets, IFN-γ level increased and the gut mucosal epithelial barrier was impaired (74, 75). The possible mechanism is that high-salt and high-fat may promote dendritic cells express co-stimulatory factor CD86 and release IL-6 and IL-1β to activate T cells and thus increase IFN-γ secretion (75). Increased IFN-γ induced internalization of tight juction proteins through macropinocytosis and led to increased permeability of the model intestinal epithelial cell line, T84 (76).

TGF-β plays an anti-proliferation role in termination phase liver regeneration (77–80). Inhibiting TGF-β in the early stage of liver regeneration may be an effective strategy to enhance proliferation and regeneration. *L. acidophilus* and *L. salivarius* have the ability to decrease TGF-β in rectum (81). However, it is reported that bone morphogenetic proteins 7, a member of TGF-β family, promote liver regeneration after PH (82). Some probiotic was found to up-regulate TGF-β (25, 83, 84). *Bifidobacterium longum* can promote peripheral blood mononuclear cells release TGF-β, while *Bifidobacterium lactis* and *Lactobacillus johnsonii* can promote epithelial cells release TGF-β (85, 86). Several *Clostridium* species produce short-chain fatty acids and thus stimulate TGF-β production by colonic epithelial cells in a TLR2, AP1–ERK pathway-dependent manner (87, 88). Also, *Clostridium* species also increase the expression of metalloproteinases at the surface of IECs, providing a large source of bioactive TGF-β within the colon. Besides, microbiota-derived products can influence the TGF-β production by lamina propria dendritic cells, such as ATP(adenosine 5′-triphosphate) increasing the expression of TGF-β in a CD70high subset of DCs of the small intestine (87). *Clostridium butyricum* can also promote the production of TGF-β by colonic lamina propria dendritic cells through TLR2 and AP1-ERK pathways (89). The up-regulation of TGF-β by gut microbiota appears to play an inhibitory role in liver regeneration, but the role of gut microbiota should be reevaluated considering the irreplaceable role of TGF-β in the termination phase.

## Gut microbiota affects liver regeneration by regulating metabolite levels

Metabolites of gut microbiota act on liver *via* gut-liver axis. Gut microbiota connects with liver regeneration mainly through its a broad range of metabolites transported through gut-liver axis. These metabolites act as important signaling and energy substrates to liver cells. The change of proportion of gut microbiota leads to change of metabolites, which may regulate liver function.

### Bile acid

Bile acids (BAs) are amphiphilic steroid molecules. As auxiliary mitogen, BAs involve in the proliferation phase of liver regeneration (7). Cholic acid (CA) and chenodeoxycholic acid (CDCA), which are the two primary BAs in humans, are synthesized from cholesterol by hepatocytes (90). In this process, Cholesterol 7α-hydroxylase (CYP7a1) is the rate-limiting enzyme. After synthesized in the liver, primary conjugated BAs are actively secreted by the hepatocyte into the biliary system. Most of the primary BAs are released into the duodenum after food intake from gallbladder. Only a few BAs return to liver through hepato-bile duct shunt.

In the upper intestinal tract, BAs help to emulsify and absorb dietary fats, cholesterol and fat-soluble vitamins. And in the lower part of the intestine (i.e. ileum and proximal colon), primary conjugated BAs undergo deconjugation and dehydroxylation by gut microbiota to form secondary BAs. In this process, the role of certain restricted clusters within the order *Clostridiales* and *Eubacaterium* is emphasized (90–92). Then, deconjugated BAs diffuse passively through the intestinal epithelium while the conjugated BAs are actively transported by the apical sodium/BAs co-transporting polypeptide in the terminal ileum (93). As a result, approximately 95% of BAs return to liver through the portal vein and this is called enterohepatic circulation of BAs. The BAs excreted in feces and urine is about 0.2~0.6g per day, which is replenished by the daily hepatic synthesis of BAs (94).

Gut microbiota has significant influence on human BAs profile (95). Gut microbiota is involved in the biotransformation of BAs through deconjugation, dehydroxylation, and reconjugation of BAs. Gut microbiota affect not only secondary BAs metabolism but also BAs synthesis in liver and affinity to BAs receptors. Gut microbiota alter the expression profile of genes involved in BAs synthesis and control key enzymes such as CYP7a1 (96). And gut microbiota inhibit BAs synthesis by alleviating FXR inhibition in the ileum (96). Besides, BAs uptake is also regulated by gut microbiota through regulating apical sodium-dependent BAs transporter and basolateral transporters (96, 97).

A study reported that the decline in gut microbiota diversity, increased pro-inflammatory *Enterococcus*, *Erysipelatrichales*, and *Enterobacteriales*, and decreased anti-inflammatory *Lactobacillus* and *Lactobacillaceae* in particular, resulted in inhibition of conversion of primary to secondary BAs and deconjugation into free BAs, which led to BAs overload and liver injury (98). Another study reported a lower secondary/primary BAs ratios in patients with cirrhosis, which may result from a increased *Enterobacteriaceae* but decreased *Lachonospiraceae*, *Ruminococcaceae* and *Blautia* (7α-dehydroxylating bacteria) abundance (90). In this report, positive correlations between CDCA and *Enterobacteriaceae*, DCA and *Ruminococcaceae*, DCA/CA ratio and *Ruminococcaceae*, LCA/CDCA ratio and *Blautia* were observed. And compared with germ-free mice, normal mice had a smaller BAs pool, with specific reductions in MCAs rather than CA (96). And positive correlation between LCA and *Parabacteroides* was observed (95).

In its basic state, BA is almost physiologically confined to the enterohepatic circulation, only allowing trace free in the systemic circulation. After PH, the capacity of remnant liver to BAs that return to liver through enterohepatic circulation suddenly decreased and this leads to BAs levels spike in systemic circulation (99–102). BAs are toxic at high levels but are also able to stimulate liver regeneration with a short term modest supplementation (103). The proper amount of BA retention could potentiate hepatocyte proliferation and induce the liver regeneration, but the excessive increase of BAs may result in liver injury (93). BAs enterohepatic circulation obstruction leads to inhibition of liver regeneration after PH (104, 105). Mice fed with cholestyramine showed decreased BAs level and hepatocyte proliferation was inhibited (106). Rats with bile external-drained showed inhibited liver regeneration after PH than rats in the control group (107). And BAs can induce the differentiation of human mesenchymal stem cells into hepatocyte-like cells *in vitro* (93). These indicate the significance of proper amount of BAs in liver regeneration.

**Figure 2:**
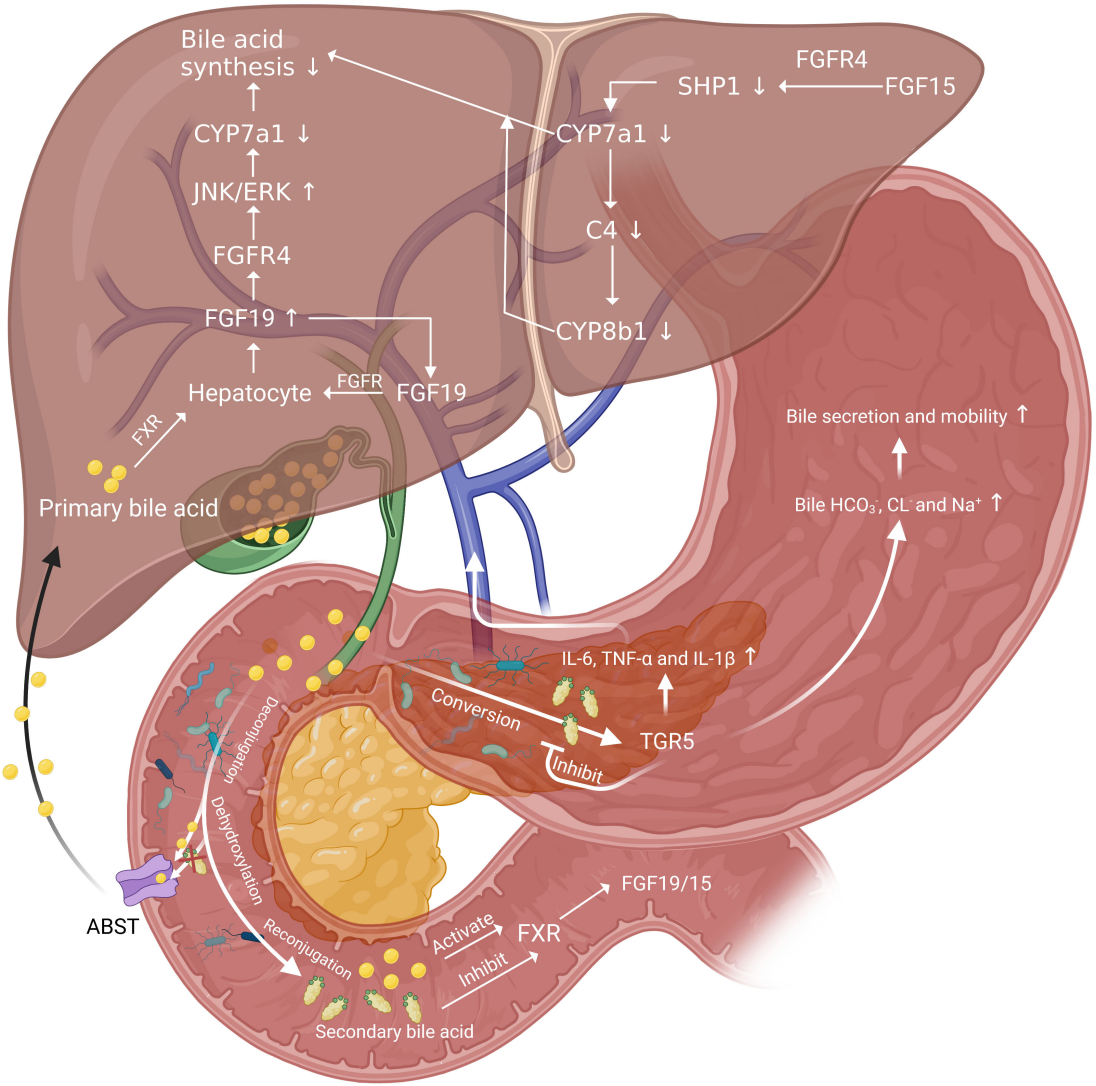
The role of gut microbiota in BAs related negative and positive regulation pathways of liver regeneration. FGF19 inhibit the rate-limiting enzyme for the synthesis of BAs, CYP7a1 and thus down-regulate BAs synthesis. And there is a positive feedback regulation of FGF19 by acting on FGFR on hepatocytes. BAs released into the intestine by the gallbladder act on FXR on intestinal epithelial cells and induce the release of FGF19 and FGF19. Then FGF19 reach liver through portal vein and function in the same way. FGF15 bond with FGFR4 to increase SHP1 expression in hepatocytes and decrease the level of CYP7a1 and CYP8b1. Secondary BAs converted from primary BAs by gut microbiota can inhibit FXR and activate TGR5, secreting inflammatory cytokines. These cytokines can be collected by portal vein and function to delay liver regeneration. The activation of TGR5 can also increase the bile secretion and mobility to reduce BAs overload in liver.

After PH, excess BAs are harmful to the liver, which may be occurred due to the biliary inflammation caused bile secretion obstruction. The biliary inflammation in mice was proved to be associated with an altered intestinal microbiome, and germ-free or antibiotic-treated mice had less pronounced liver disease compared with conventionally housed mice (108). However, there are several protective mechanisms that mitigate BAs damage to the liver. Increased secretion of bicarbonate in bile mitigates the damage of large BAs by regulating PH (109). When BAs are in excess, a negative feedback regulation initiated by nuclear receptors farnesoid X receptor (FXR) can effectively inhibit BAs synthesis. FXR is highly expressed in the liver and ileum, which can regulate the expression of some important cell cycle transcription factors, such as Foxm1b and cyclin D1 (18). FXR senses BAs (mainly primary BAs like CA and CDCA) and is activated in the ileum and liver, and then fibroblast growth factor 19 (FGF19; FGF15 in mouse) is released. FGF19 is collected by portal vein system and binds to hepatic FGF receptor 4/Klotho-β cell-surface receptor complex, inhibiting CYP7a1, the rate-limiting enzyme for the synthesis of BAs, by activating JNK 1/2 and extracellular signal regulated kinase 1/2 (21, 110–112). Besides, the activation of FXR can induce expression of small heterodimer partner 1, leading to inhibition of CYP7a1 synthesis (113). The decrease of CYP7a1 synthesis inhibited the rate of BAs synthesis and thus protect liver (**Figure 2**).

Obeticholic acid, a selective FXR agonist, was reported beneficial for improving liver injury and promoting liver regeneration (114, 115). Compared with wild-type mice, FXR deficient mice showed accumulation of primary BAs, increased levels of *Bacteroidetes* and decreased levels of *Firmicutes* after high fat diet for 10 weeks (116). Brain-dead induced intestinal damage and down-regulate FXR, and this led to reduction in intestinal FGF 15, liver damage and regenerative failure (117).

In addition to FXR, the secondary BAs activate G-protein-coupled receptors, and G-protein-coupled receptor 5 (TGR5) also plays an important role in protecting the liver from BAs overload. TGR5 can be found in multiple parts of the body. In the liver, TGR5 mainly exists on the surface of bile duct cells, endothelial cells, and Kupffer cells, but there is little or even no expression on the surface of liver cell membrane (118, 119). And it is reported that FXR can bond to the responsive element on the TGR5 gene promoter and induce the transcription of TGR5 (120). Currently, four possible related mechanisms of TGR5 protecting the liver are proposed. Firstly, TGR5-KO mice showed more hydrophobic BAs composition than control group both before and after PH (101), which suggests TGR5 may work by altering the composition of BAs pools, specifically by inhibiting the conversion of BAs to hydrophobicity. Secondly, such cytokines as IL-6, TNF-α and IL-1β elevate in plasma significantly in TGR5-KO mice after PH (101), which may have contributed to cause inflammation and delay liver regeneration. Therefore, TGR5 may modulate the production and release of these cytokines after PH. Thirdly, TGR5 may control adaptive ion transport in bile when BAs overloaded after PH. Bile flow and ${\text{HCO}}_{3}^{-}$ , CL^-^ and Na^+^ increased in wild mice 48h after PH. Among that, the ${\text{HCO}}_{3}^{-}$ secretion is mainly mediated by Anion exchange 2 (AE2), sodium-independent Cl−-HCO3− anion exchanger and the major AE protein expressed in biliary epithelial cells (121). However, such changes were not observed in TGR5-KO mice (101). The TGR5-dependent increase of bile ${\text{HCO}}_{3}^{-}$ and Cl^-^ output after PH may enhance bile secretion and bile mobility and thus protect the liver from BAs overload (122, 123). Finally, TGR5 may contribute to the elimination of BAs in urine through control MRP2 and MRP4 gene expression in the kidney (101, 118).

### Lipopolysaccharide

Lipopolysaccharide (LPS) is the major component of the outer wall of the cytoderm of Gram-negative bacteria, which is made up of lipid A, O-antigen and core oligosaccharide. There is a large number of Gram-negative bacteria in human gut, such as *Escherichia coli*, *Proteus* and *Pseudomonas aeruginosa*. When these bacteria die, LPS will come off by dissolving and destroying cells, and exert its toxicity by acting on human cells.

**Figure 3:**
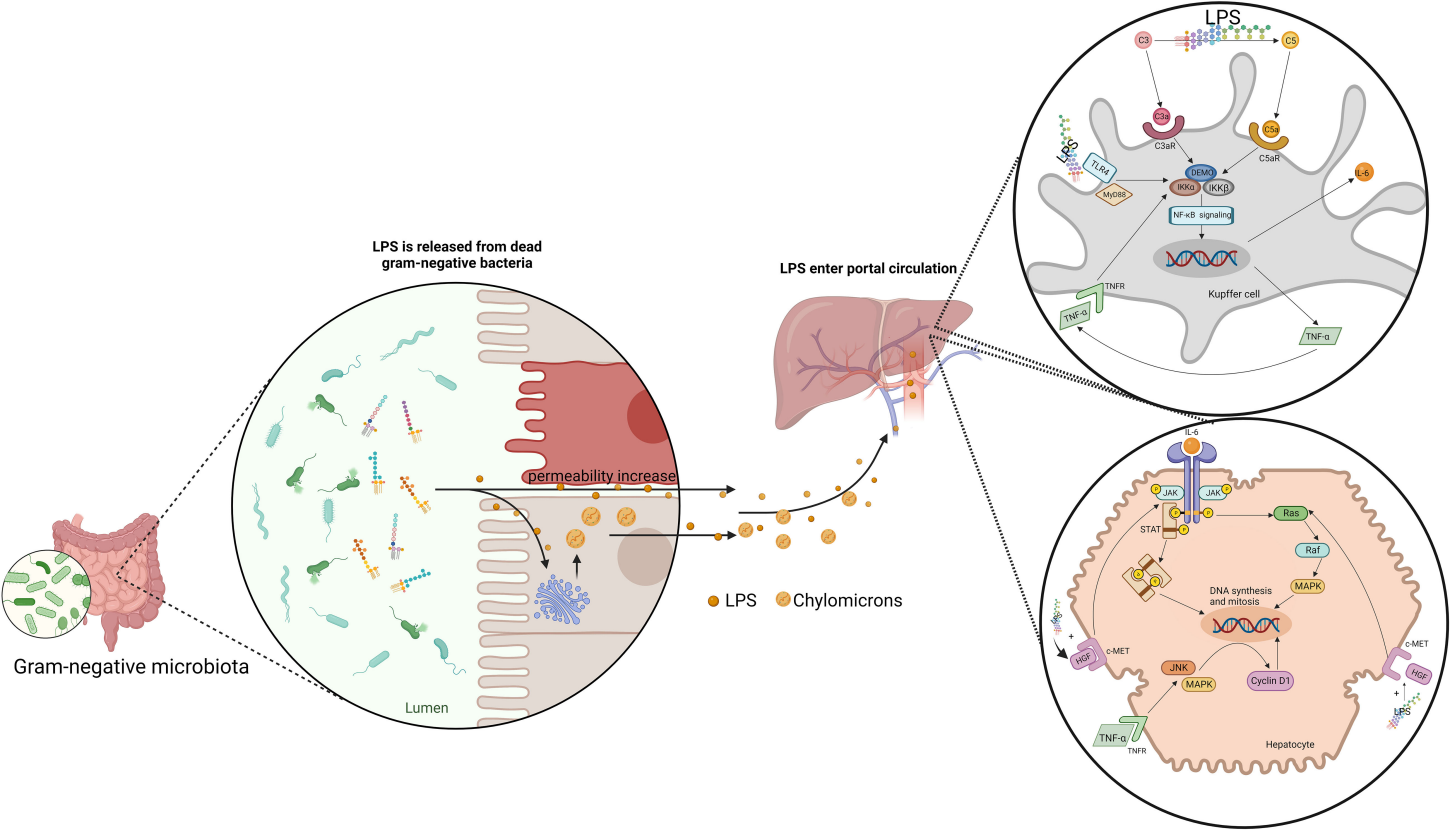
Gut-derived LPS translocates by chylomicrons and increase intestine permeability to promote liver regeneration. LPS induce the release of IL-6 and TNF-α in Kupffer cells through NF-κB signaling pathway. IL-6 promote the DNA synthesis and mitosis of hepatocytes by activating JAK/STAT pathway and Ras-Raf-MAPK pathway. IL-6 promote the DNA synthesis and mitosis of hepatocytes by activating JAK/STAT pathway and Ras-Raf-MAPK pathway. TNF-α activate JNK and MAPK-ERK, inducing cyclin D1 expression. Besides, LPS can enhance the effect of HGF on hepatocytes.

Just as BAs, the right amount of LPS is beneficial to liver regeneration. Such hepatotrophic factors as EGF and insulin were observed to be released in large quantities in mice with LPS administered both before and after PH, which promoted hepatocyte DNA replication and liver regeneration (124). The biliary epithelial cells loaded with LPS are able to activate MAIT cells in an MR1-dependent manner, suggesting an immune surveillance effector response against invading bacteria in the human liver, which facilitate liver regeneration (125). Eliminating gut microbiota, LPS and other means of limiting the right amount of LPS translocating inhibited hepatic DNA synthesis in mice (126). And the same was observed in LPS-resistant rats, Gram-negative bacteria deficient rats, LPS-resistant mice and mice with simultaneous resection of the bowel and PH (126–128). LPS supplementation can reverse delayed liver regeneration (126, 127). Besides, LPS can stimulate the potential of Kupffer cells to promote liver regeneration. LPS lead to the classical activation of Kupffer cells by binding to TLR-4 (129). Kupffer cells have been proved to be beneficial to liver regeneration (129, 130). Activated Kupffer cells release TNF-α and IL-6, which are the key factors in the priming phase of liver regeneration (129, 131). Moreover, Kupffer cells promoted liver regeneration by affecting liver progenitor cells (130, 132). Kupffer cells depletion inhibited liver progenitor cells differentiation into hepatocytes. Finally, LPS can enhance the promoting effect of HGF on hepatocyte proliferation. HGF can induce the JNK and AP-1 DNA binding activity, which is beneficial to liver regeneration. A higher level of the binding activity was observed after combining the LPS and HGF compared with HGF alone (133) (**Figure 3**).

Excess LPS can also lead to various types of liver injury. For example, long-term alcohol consumption leads to increased intestinal permeability, which increases LPS levels and causes alcoholic liver disease (134). And LPS can be involved in acute liver injury as a cofactor (135). Intravenously administered glutamine after hepatectomy reduces bacterial and LPS translocations and significantly improves liver regeneration (136). Therefore, whether LPS is beneficial to liver regeneration or aggravating liver injury may depend on its amount and duration of exposure.

### Short-chain fatty acids

Short-chain fatty acids (SCFAs), with up to six carbon atoms in length, are the principle end products of carbohydrates and proteins metabolized by gut microbiota. Acetate, propionate, and butyrate, are the principal SCFAs in the human gut (137). These SCFAs can be synthesized in the gut through a variety of pathways (138). And *Bacteroidetes* are the primary producers of acetate and propionate, which *Firmicutes* are the primary butyrate producers (139). Many factors, such as gut microbiota composition and the amount of dietary fiber in the diet can modulate the level of SCFAs in the gut (138, 140).

SCFAs promote liver regeneration by maintaining the integrity of gut mucosal epithelial barrier and improving the metabolism homeostasis. SCFAs enhance the gut mucosal epithelial barrier through providing energy and modulating immunity. The content of SCFAs decreased with intestinal extension, high in cecum and proximal colon but low in the distal colon (141). This indicates SCFAs act as an energy source for colon cells. Actually, acetate, propionate, and butyrate are consumed by epithelial cells for energy. And energy source provides more than half of the energy required by epithelial cells of the distal colon (137). This energy supply increases the synthesis of mucin and mucosal lipids and improve the tight-junction of the gut mucosa (137, 142, 143). Acetate is the key metabolite of *Bifidobacteria* to inhibit enteropathogens (144). In addition, SCFAs are significant extracellular agonists for some G-protein-coupled receptor (GPR). Acetate, propionate and butyrate can activate GPR41 and GPR43 in the epithelium while butyrate can also activate GPR109A (138). Activated GPR43 plays a role in modulating regulatory T cells (145, 146). And GPR109A activated by butyrate promotes the differentiation of regulatory T cells and IL-10-producing T cells (147). Also, butyrate is important in the peribiliary fibroproliferative responses and can temper the induced fibrosis, which is beneficial to bile secretion and liver regeneration (148). Except being an extracellular agonist, butyrate and propionate can inhibit the activity of histone deacetylases in colon cells and immune cells, and thus modulate the differentiation of regulatory T cells (149–151). Acetate was also found to inhibit histone deacetylases in activated T cells (152). Regulatory T cells differentiate into different cells under the effect of SCFAs to induce anti-inflammatory effects, which play a significant role in maintaining gut mucosal epithelial barrier. And normal colon cells rely primarily on butyrate as an energy source and reduce the level of butyrate, but cancer cells become less dependent on SCFAs due to metabolic reprogramming, which means a higher level of inhibitory effect of histone deacetylases and inhibits proliferation in cancer cells (138). The intact barrier limits bacterial and LPS translocation and facilitates liver regeneration. Besides, activated GPR41and GPR43 can activate extracellular signal-regulated kinases 1/2, JNK, and p38/mitogen-activated protein kinase (153). Recent reports have revealed the potential value of JNK1 in liver regeneration (6). The active form of JNK1 was upregulated 1 hour after ALPPS and this might lead to the release of a ligand named Indian hedgehog (IHH) inducing hedgehog signaling, from stellate cells (6). The level of IHH downstream factor GLI1 and its proliferative target CCND1 elevated later (5, 6). CCND1 encodes cyclin D1, which is a crucial promoter of the hepatocyte cell cycle (5, 154). Therefore, SCFAs may promote JNK1-IHH-GLI1-CCND1 pathway through GPR and thus improve liver regeneration.

In addition, SCFAs can improve the metabolism homeostasis during liver regeneration. SCFAs that are not oxidized by colon cells reach the liver through the portal vein. Propionate can induce the hepatic gluconeogenesis. Besides, SCFAs increase the postprandial release of gut hormone GLP-1 and peptide YY, improving the metabolic phenotype (155, 156).

### Other metabolites

Hydrogen (H_2_) production takes place in the human gut. However, mammalian cells cannot produce hydrogen on its own because it lacks the key hydrogenase. H_2_ in human gut is mainly produced by anaerobic bacteria, such as *Firmicutes* and *Bacteroides* (157). And pyruvate is one of the most important substrate for H_2_ production (137). The antioxidant activity of H_2_ can improve intestinal inflammation and enhance the gut mucosal epithelial barrier (158). Endogenous H_2_ production induced by lactulose promoted liver regeneration after 70% PH in rats (159).

Indoles and indole derivatives produced by gut microbiota metabolizing tryptophan in the gut also play a role in promoting liver regeneration through maintaining the gut mucosal epithelial barrier. Indoles up-regulate the expression of genes involved in maintaining tight junctions in epithelial cells (160, 161). Indole acetic and indole-3-propionic acid can affect the gut mucosal epithelial barrier integrity through the activation of AhR or PXR transcription factors (162, 163). Goblet cell failure leading to decreased mucus secretion is an important manifestation of intestinal inflammation. And indoleacrylic acid can promote the differentiation of goblet cell and mucus production (164). Indoleacrylic acid and indole-3-propionic acid can enhance the production of anti-inflammatory cytokine in epithelial cells and macrophages, while indole-3-propionic acid and indole acid can inhibit the pro-inflammatory cytokine in macrophages and hepatocytes (164, 165). This indicates indole and its derivatives may affect immune response in the liver and thus affect liver regeneration, but further research is needed to explore the mechanisms and confirm the idea.

## Methods of regulating gut microbiotics promote regeneration

The liver regeneration effect of gut microbiota exists both in physiological and pathological effect. In the basic medicine researches, PH are the common methods of building models to investigate direct hyperplasia related liver regeneration. And the conclusions from the studies conducted in the physiological condition may be universally appropriate in different conditions. However, in terms of the researches in specific liver disease intervention methods, most of the researches are conducted related in the particular pathological condition. Much evidence have revealed that liver injury can significantly inhibit the normal program of liver regeneration through excess inflammation, scarring and epithelial abnormalities, such as fatty liver disease, chronic scarring, prior chemotherapy and massive liver injury, so the conclusions from pathological conditions only can be appropriate in what we have discoveried (166).

Multiple methods, such as probiotics, prebiotics and antibiotics can be used to modulate the proportion of gut microbiota and thus, promote liver regeneration. From this point of view, we summarize the discoveries of methods of modulating gut microbiota in PH liver injury models to promote liver generation and expand the discussion to promoting regeneration and repair of the failing liver in specific disease.

### Dietary factors

There are also factors that regulate gut microbiota and thus affect liver regeneration. The gut is the place where food is digested, so the influence of dietary factors on gut microbes cannot be ignored. Malnutrition is likely to lead to translocation of gut microbiota and its metabolites (167). However, some dietary components with anti-inflammatory or antioxidant properties can modulate the gut microbiota (168). Protein, vitamin and fish-oil-supplemented diets are helpful for alleviating liver ischemia reperfusion injury, while folic acid and vitamin improve alcoholic liver disease (83). It is reported that vitamin D receptors play an important role in the composition of gut microbiota in mice (95). Mice fed with fish-oil showed ameliorated liver injury by faster restoration of serum alanine aminotransferase (ALT) and total bilirubin (TBIL) levels and accelerated liver regeneration after 70% PH (169). And rosa mosqueta oil intake demonstrated ALA, PEA and DHA increase in liver, promoting liver regeneration (170). Arginine increased the hepatic catabolism functions, but was unable to confirm its benefits in liver regeneration in rats (171). Glutamine can promote hepatic alanine uptake and intestinal glutamine metabolism, reduce bacterial and LPS translocation, and thus significantly improves the mitoses of hepatocytes 72h after 60% PH (136, 172). Lipid emulation administration is useful in liver transplantion from both the steatotic or non-steatoic liver donor, contributing to intestinal microbiota preservation and liver regeneration (173). And patients supplied with BCAAs administered two times a day for six months after PH showed the liver uptake value three-folds higher than the control group, which indicated improved liver functionality and accelerated regenerative capacity (174). Besides, ankaflavin, a traditional food additive used in Eastern Asia and China, significantly reduce the apoptosis of hepatocytes (175). Also, Korean red ginseng extract, which contains ginsenosides, phenolic compounds, polysaccharides, and polyacetylenes, showed a chemopreventive effect of preventing hepatocytes apoptosis (176). An enhanced diet with vitamins C and E and supplemented with polyphenols also can reduce the hepatocellular damage.

As for the pro-regenerative effect of dietary factor in liver pathological condition, mice with a high-fat diet showed a reduction in the ratio of *Firmicutes* to *Bacteroidetes* and decreased gut microbiota richness, while mice with low-fat diet promoted *Firmicutes* (177). In mice with a high-fat diet, hepatic PPARγ expression is increased and liver regeneration is inhibited (178). Retinoic acid intake can regulate lipid homeostasis and promote liver regeneration, which is associated with a reduction in the ratio of *Firmicutes* to *Bacteroidetes* (155, 179). Mice fed with alcohol showed increased *Actinobacteria* and *Proteobacteria*, and an increased ratio of *Firmicutes* over *Bacteroidetes* (180, 181). And this may lead to bacterial overgrowth, increased intestinal permeability and translocation of microbiota and LPS (182). UDCA administration has been approved to be a clinical intervention of primary biliary cirrhosis (PBC) to improve liver injury and promote liver regeneration. And administration of nor-UDCA are also approved treatments to improve liver injury in patients with sclerosing cholangitis (183). Bovine colostrum and Zinc were reported to enhance the gut mucosal epithelial barrier and inhibit the translocation of microbiota and LPS (184, 185). Besides, the effect of starvation before PH on liver regeneration is still controversial.

### Probiotics

Probiotics are defined as “monocultures or mixed culture of live microorganisms that, when administered in adequate amounts, confer a health benefit on the host by improving the properties of his own microflora, which is microbial, viable and beneficial to health (186, 187)”. Probiotics have been reported to be favorable to ameliorate liver injury and motivate liver regeneration in recent years. For example, N. Rayes, et al. reported a specific synbiotic composition of pre- and probiotics is related to the increase of liver functional capacity measured by LiMAx (188). The mechanism promoting liver regeneration consists of stabilize the gut mucosal epithelial barrier and prevent bacterial translocation, modulate the level of cytokines and inflammatory factors and affect functions of a variety of immune cells. And the essence of these mechanisms is to modulate the proportion of microflora in the gut.

Probiotics intakes modulate the proportion of gut microbiota, and the proportion of gut microbiota affects many factors related to liver regeneration, including the integrity of gut mucosal epithelial barrier, the secretion of pro and anti-inflammatory cytokines exposed to liver. Xuelong Li, et al. found a significant increase in the amount of *Lactobacillus* and *Bifidobacterium* in ALD patients after supplementation of Lactobacillus casei (189), which improve lipid metabolism in liver and liver injury in ALD condition. Jun Li, et al. tested function of a novel probiotic mixture Prohep, which is composed of *Lactobacillus rhamnosus GG* (LGG), viable *Escherichia coli Nissle 1917* (EcN), and heat-inactivated VSL#3 (1:1:1) and founded increased beneficially anti-inflammatory bacteria and decreased Th17-inducing bacteria (190). Moreover, this study also showed increased *Bacteroidetes* levels and decreased *Firmicutes* and *Proteobacteria* levels in mice with HCC (190), and this suppressed HCC growth. Probiotics also up-regulate SCFAs-producing bacteria, and thus increase the level of SCFAs transported into liver (190, 191). Laetitia Rodes, et al. also founded *Bifidobacterium longum* subsp. *infantis* ATCC 15697 impeded the growth of endotoxins-producing bacteria (191). Janelle C. Arthur, et al. reported that the 8-strain preparation VSL#3 significantly decreased the abundance of *Bacteroidetes* and *Clostridium* bacteria and stimulate the TGF-β secretion (192).

In many pathological processes, large amount of LPS and bacterium translocation is increased with the increased permeability of gut mucosal epithelial barrier, and this leads to numerous liver injury and inhibits liver regeneration. Many studies reported that probiotics can enhance gut mucosal epithelial barrier through multiple mechanisms. VSL#3 pretreatment protected gut mucosal epithelial barrier, reduced bacterial translocation in a mouse model of sepsis (70, 193). Yuhua Wang, et al. reported *Lactobacillus rhamnosus GG* enhanced tight junction of intestinal mucosa epithelial cells and decreased epithelial cell permeability in a mouse model of ALD (194).

One mechanism is that probiotics affects function of a variety of immune cells *Lactobacillus rhamnosus GG* has been shown to enhance this barrier by up-regulating EGF-R, intestinal mucins, heat shock protein (HSP25 and 27) and its receptors (25). And immune cells like neutrophil (83, 195) are modulated by *Lactobacillus casei Shirota* and CD8^+^ T cells (25) by *E. coli, Salmonella typhimurium or Clostridium difficile* to prevent and bacterial translocation.

Inflammation is another factor that increase permeability of gut mucosal epithelial barrier. Multiple cytokines are involved in the occurrence of intestinal inflammation. Probiotics regulate a wide variety of cytokine, and the effect may be mediated by altering the balance between proinflammatory and anti-inflammatory or regulatory cytokines (196). A number of study reported that *Lactobacillus acidophilus*, *Bifidobacterium longum and Lactobacillus salivarius* can up-regulate anti-inflammatory cytokine IL-10 and TGF-β (25, 49, 83, 84). The down-regulation of proinflammatory cytokines can be also done by probiotics, such as IL-1β (48, 195) by *Lactobacillus casei Shirota*, IL-6 and IFN-γ (25, 46, 48, 49, 70, 71) by VSL#3 probiotics compound and *Parabacteroides distasonis*, IL-8 (48, 49) by *Lactobacillus acidophilus* and *Bifidobacterium longum*, IL-12 (71) by *Parabacteroides distasonis*, IL-17 (71, 195) by *Lactobacillus casei Shirota*, TNF-α (25, 46, 48, 49, 70, 83, 197, 198) by *L. acidophilus*, *L. rhamnosus*, *L. paracasei*, *L. plantarum P. pentosaceus*, *B. lactis*, *B. breve* and *S. thermophilus*. The regulation of cytokine levels reduces the risk of inflammation, and thus enhances the gut mucosal epithelial barrier.

In summary, Probiotics can prevent LPS and bacterial translocation by stabilizing the integrity of gut mucosal epithelial barrier through multiple mechanisms, therefore, liver injury is ameliorated and thus promotes liver regeneration. Besides, Probiotics are noticed to reduce ALT, AST, GGT and ALP in NAFLD patients, which are the most widely used biochemical indicators of liver cell injury (46, 48, 193, 199). It is worth noting that most of the current studies are focus on the probiotic treatment in pathological. The direct pro-regenerative effect of probiotics needs to be further unrevealed.

### Prebiotics

In addition to providing probiotics directly, the provision of growth substrates for gut microbiota to induce compositional or metabolic changes is also a strategy. Prebiotics refer to indigestible food components but promote the growth and activity of some beneficial gut microbiota, thus producing beneficial physiological effects on the host (200).

Current established prebiotics include fructans, dietary polyphenols, oligofructose, inulin, fructooligosaccharides, lactulose, galactan, galactooligosaccharides, resistant starch, pectin and milk oligosaccharides (201, 202). Specific prebiotics have been shown to improve such gut microbiota as *Lactobacillus*, *Bifidobacterium*, *Faecalibacterium prausnitzii* and *Akkermansia muciniphila* (201, 203). Prebiotics improve gut mucosal epithelial barrier function, induce the secretion of mucus and immunoglobulin A, improve intestinal motility, and prevent the colonization of pathogenic bacteria in the gut, to reduce translocated entrogenous pathogen into liver (117). Prebiotics reduced infection rates after liver transplantation (204). And inulin can prevent liver cancer through its anti-angiogenic properties and reducing pro-inflammatory pathways (205, 206). It is reported that prebiotics significantly reduced TG, TC, LDL-C, ALT, AST, and GGT in patients with NAFLD (199). Lactulose induced endogenous H_2_ production and accelerate liver regeneration after 70% PH in rats (159). Fructose protect hepatocytes from TNF-induced mitochondrial apoptosis through activating the JNK pathway (207). However, high fructose intake can disrupt gut microbiota homeostasis and fill hepatocytes with fructose-1-phosphate, leading to acute ATP depletion and adverse liver regeneration (207). Therefore, appropriate prebiotics can promote the stability of the gut microbial community and is beneficial for gut microbiota homeostasis, indicating its potential in improving liver injury and promoting liver regeneration.

However, current studies about prebiotics have put most of the importance on the gut microbiota proportion restoration effect of prebiotics, but how the gut dysbiosis recovery function is still need to further study on.

### Fecal microbiota transplantation

Fecal microbiota transplantation (FMT) is a new strategy to restore healthy intestinal flora. FMT refers to transplant the functional flora from healthy human feces into the patient’s gut (208). Antibiotics can eliminate some Gram-negative bacteria in the gut, reducing endotoxin release, but may also destroy some beneficial gut microbiota. Auto-FMT can reestablish the microbiota after damage caused by antibiotics and normalize the damage program of liver regeneration (27, 209). In this study, *Lachnospiraceae*, *Ruminococcaceae*, and *Bacteroidetes* were successfully reestablished after auto-FMT (209). Also, FMT gavage treatment can improve the liver lobules injury and promote them developing towards normal tissue (210). Besides, FMT can also reduce the expression of gut microbial antibiotic resistance genes in patients with cirrhosis (211). And severe alcoholic hepatitis patients treated with FMT have an improved survival rate (114). In patients receiving hepatic resection, preoperative and postoperative FMT improve liver injury and reduce complications (212). FMT can modulate the gut microbiota and maintain intestinal homeostasis more directly. It is probably to regulate the composition of gut microbiota accurately, increase the proportion of bacteria beneficial to liver regeneration, prevent the pathological bacteria translocation and control the level of BAs and LPS within a beneficial range to improve the mitosis in liver (213). More research is needed before we can achieve the goal indeed.

### Immunosuppressive agents and antibiotics

Immunosuppressive agents and antibiotics can also affect gut microbiota and thus affect liver regeneration. Cyclosporine A restored the gut microbiota after liver transplant and improved liver injury (214). Tacrolimus increased probiotics and decreased endotoxin-producing bacteria, which is beneficial for functional recovery and regeneration after liver transplantation (215). Antibiotics can reduce the total number of gut microbiota, eliminate bacteria that have a high ability to translocate, but they can also damage some beneficial gut microbiota. Polymyxin B sulfate reduced *Enterobacteriaceae*, and increased *Bifidobacterium*, *Lactobacillus*, *Bacteroidetes* and *Eubacterium* in rats, and this lead to reduction in LPS and TNF-α (216). Rifaximin, neomycin, erythromycin, ampicillin-sulbactam, metronidazole, vancomycin and norfloxacin have been reported to improve liver injury in a variety of liver diseases and surgeries, but the role of promoting liver regeneration needs to be further studied (117, 217). However, oral ampicillin killed some liver regeneration related commensal bacteria and inhibited liver regeneration (218).

## Discussion

Gut microbiota associated with liver regeneration through various pathways. In this review, we summarized the dynamic change of the gut microbiota composition after PH, which mainly involved several subspecies of *Bacteroidetes* and *Firmicutes.* And the review demonstrated the role of metabolites and cytokines in different phases of liver regeneration and how gut microbiota played a role in these processes.

Many previous studies have focused on the mechanism of gut microbiota in improving various kinds of liver diseases including ALD, NAFLD and so on, but there is little attention has been paid on how gut microbiota promote liver regeneration. Our review concerning the role of gut microbiota in the process of liver regeneration elucidated the mechanisms by which gut microbiota influences liver regeneration through cytokines and metabolites and filled the gap in this field.

Gut-derived pathways in liver regeneration described in our review are mainly based on animal models, which still needs to be confirmed in future clinical research. Besides, changes of gut microbiota composition when liver regeneration observed in different studies were not entirely consistent and even opposite. More related studies are needed to find out how gut microbiota changes. And the regulation of gut microbiota on cytokines and metabolites conducive to liver regeneration remains to be further studied in much detail.

In summary, gut microbiota promote liver regeneration mainly through cytokines and its metabolites. Furthermore, we provide strategies for altering the composition of gut microbiota in favor of liver regeneration. We hope that our review will provide a theoretical basis for future clinical application of gut microbiota to promote liver regeneration and improve the quality of life of the patients with liver disease.

## Author contributions

Writing – Original Draft, ZX and NJ. Visualization, ZX. Writing – Review and Editing, ZW, KY, and YX. Supervision, Z.W. All authors contributed to the article and approved the submitted version.

## Funding

This work was supported by grants from the Science and Technology Major Program of Sichuan Province (2022ZDZX0019), and the Natural Science Foundation of China (82270643, 82170621, 82070644, 81800564 and 81770615).

## Acknowledgments

The illustrations are created with BioRender.com.

## Conflict of interest

The authors declare that the research was conducted in the absence of any commercial or financial relationships that could be construed as a potential conflict of interest.

## Publisher’s note

All claims expressed in this article are solely those of the authors and do not necessarily represent those of their affiliated organizations, or those of the publisher, the editors and the reviewers. Any product that may be evaluated in this article, or claim that may be made by its manufacturer, is not guaranteed or endorsed by the publisher.
